# The Eye

**Published:** 1869-04

**Authors:** 


### The Eye.

### ARRANGEMENT OF LIGHTS IN CHURCHES, THEATRES AND PUBLIC HALLS.

The arrangement of light in chuches, theatres, and assembly rooms; the destruction of the oxygen in consequence of its consumption by the assembly, and by the numerous gas lights, as well as by the necessity of facing a bright, glaring light, in order to see the speaker, are serious causes of derangement of vision.

Our public rooms should all have higher ceilings, be better ventilated, and the light so arranged that the eyes of the audience are less exposed to its glare. It is an easy matter to shade the pulpit lights, so that the minister would be afforded sufficient light for his manuscript, and still his auditory be relieved from looking it directly in the face during the entire period of service. Mr. Beecher has an arrangement in his church for lighting his manuscript that it would be well for other ministers to adopt. Two little gas jets are arranged on either side of the desk, with black enameled shades next the audience, so that his congregations' eyes are protected from the glaring light usually employed in other churches.

When more care is taken to make churches healthy places, in which to collect large congregations, to give them air to breathe as well as places to sit, and when glaring white walls—glass, so stained as to tint objects with every hue of the rainbow—and gas lights so placed as to shine naked, in the eyes of the worshippers, shall be abandoned, then shall we consider it our *duty* and a pleasure to attend church, and not until then.

The lights should be provided with globes of blue tint—the walls should be frescoed with dark colors and without the usual display of 'dutch metal" or gold—and *inside* shades should be provided for the long, stained glass windows. Churches are frequently made too dark and prison like in the day time; this should also be avoided, for the class whose eyes are feeble and sensitive is very large, and their eyes are subjected to a dangerous test when emerging from a badly lighted room to the clear sunlight of day. Indeed it is not unfrequently the case that complete "nervous blindness" is caused in this manner. To avoid this danger, Eastern people are in the habit of anointing their eye lashes with a pigment made of antimony and oil, and Artic Voyagers employ coverings to the eyes through which are made very small apertures for the admission of the intense light.

Light is a natural stimulus to the eye, and it requires it; but the reading of finely printed novels, double-column books, and diamond bibles by artificial light of any kind, is a very pernicious habit.

